# NLRP1 polymorphisms in patients with asbestos-associated mesothelioma

**DOI:** 10.1186/1750-9378-7-25

**Published:** 2012-10-02

**Authors:** Martina Girardelli, Iva Maestri, Rosa R Rinaldi, Mauro Tognon, Renzo Boldorini, Massimo Bovenzi, Sergio Crovella, Manola Comar

**Affiliations:** 1 Institute for Maternal and Child Health–IRCCS “Burlo Garofolo”, Trieste, Italy; 2 Institute for Maternal and Child Health-IRCCS “Burlo Garofolo”, University of Trieste, Trieste, Italy; 3 Department of Experimental and Diagnostic Medicine, Pathology Unit of Pathologic Anatomy, Histology and Cytology University of Ferrara, Ferrara, Italy; 4 Deartment of Laboratory Medicine, Operative Unit of Anatomy-Pathology, Sant’Anna University Hospital of Ferrara, Ferrara, Italy; 5 Section of Cell Biology and Molecular Genetics, School of Medicine and Surgery, University of Ferrara, Ferrara, Italy; 6 Department of Medical Sciences, Faculty of Medicine, University of the Piemonte Orientale, Novara, Italy; 7 Clinical Unit of Occupational Medicine, University of Trieste, Trieste, Italy

**Keywords:** Mesothelioma, Asbestos, Inflammasome, NLRP1, NLRP3

## Abstract

**Background:**

An increasing incidence of malignant mesothelioma (MM) cases in patients with low levels of asbestos exposure suggests the interference of alternative cofactors. SV40 infection was detected, as co-morbidity factor, only in 22% of asbestos-MM patients from a North-Eastern Italy area. An additional mechanism of injury related to asbestos exposure in MM development has been recently associated to inflammatory responses, principally driven by interleukin (IL)-1 beta (ß) activated within the inflammasome complex.

*NLRP3* inflammosome has been described as the intracellular sensor for asbestos able to induce inflammasome activation and IL-1ß secretion while *NLRP1* is expressed in lung epithelial cells and alveolar macrophages and contributes to the immune response and to survival/apoptosis balance. This study proposes to evaluate the impact of known *NLRP3* and *NLRP1* polymorphisms in the individual susceptibility to asbestos-induced mesothelioma in subjects from a hyperendemic area for MM.

**Methods:**

134 Italian patients with diagnosis of mesothelioma due (MMAE, n=69) or not (MMAF, n=65) to asbestos, 256 healthy Italian blood donors and 101 Italian healthy subjects exposed to asbestos (HCAE) were genotyped for *NLRP1* (rs2670660 and rs12150220) and *NLRP3* (rs35829419 and rs10754558) polymorphisms.

**Results:**

While NLRP3 SNPs were not associated to mesothelioma, the NLRP1 rs12150220 allele T was significantly more frequent in MMAE (0.55) than in HCAE (0.41) (p=0.011; OR=1.79) suggesting a predisponent effect of this allele on the development of mesothelioma. This effect was amplified when the *NLRP1* rs2670660 allele was combined with the *NLRP1* rs12150220 allele (p=0.004; OR=0.52).

**Conclusion:**

Although *NLRP3* SNPs was not involved in mesothelioma predisposition, these data proposed *NLRP1* as a novel factor possibly involved in the development of mesothelioma.

## Background

The multistage evolution of pleural malignant mesothelioma (MM) is characterized by the occurrence of pathogenetic events involving tumorigenic agents and, among these, the exposure to asbestos fibers results as the main risk factor [1]. Recently, the observation of an increasing incidence of mesothelioma cases in patients with low levels of asbestos exposure suggests a more complex process probably due to the interference of alternative cofactors. (i.e. radiation, other mineral fibers, genetic and viral ones) involved in a complex pathogenic process whereby genomic damage mechanisms work over a long latency period [2-6].

Recent studies emphasize the role of the Simian Virus 40 (SV40) and asbestos as co-carcinogens in causing mesothelioma, lowering the asbestos threshold limit and the latency period of carcinogenesis [7-14]. Nevertheless, the broad variability (0% to 100%) in the detection of SV40 strain in the different cohort of MM affected patients showed a controversial role of this virus as causative agent.

In Italy, the vast asbestos consumption is generally related to occupational origin and restricted to some geographic area characterized by a higher incident rate of MM. The contributory role of SV40 infection in MM patients characterized by an “ascertained” asbestosis from an north eastern Italy area hyperendemic for MM, with a standardized incidence rates 4 time higher than the national rate (standard population: Italy census, 2001), showed that SV40 were detected in 22.2% of the patients, but the data was not found statistically significant [15].

An additional mechanism of injury related to asbestos exposure in MM development has been recently associated to inflammatory responses, principally driven by interleukin (IL)-1 beta (ß) activated within the inflammasome complex [16].

An important role for IL-1ß has been proposed in the pathogenesis of asbestos-induced mesothelioma because it regulates human mesothelial cell proliferation [17], and IL-1ß–driven inflammation is well known to promote the development and invasiveness of several tumor types in vivo [18].

The study by Dorset et al. [19] supports the implication of the Nod-Like Receptor Pyrin domain containing 3 (*NLRP3)* inflammasome complex in the pulmonary inflammatory diseases linked to asbestos and air pollutants. In addition, polymorphisms in *NLRP3* gene have been recently associated with chronic inflammatory diseases, autoimmune disorders and infections [5,6,10,20,21]. Moreover other two SNPs in *NLRP1*, a gene that codifying for another inflammasome receptor, have been recently reported to contribute to the immune response in lung epithelial cells and alveolar macrophages [14].

Taking in account the previously reported association between variants in *NLRP3* gene and an impaired IL-1 production as well as the role of IL-1 driven inflammation in asbestos lung fibrosis and mesothelioma, the aim of this study was to evaluate the impact of the *NLRP3* and *NLRP1* polymorphisms in the individual susceptibility to asbestos-induced mesothelioma in a cohort of MM patients from a North-Eastern Italy area hyperendemic for malignant pleural mesothelioma. For comparison a series of healthy subjects from the same area at higher risk for environmental and domestic exposure to asbestos and a group of healthy subjects not exposed to asbestos were included as reference control groups.

## Results

We analyzed *NLRP3* rs35829419, rs10754558 SNPs and *NLRP1* rs2670660, rs12150220 SNPs in 134 Italian patients with mesothelioma, in 256 healthy controls and in 101 individuals exposed to asbestos with no mesothelioma at the age of the enrollment. SNPs frequencies were in Hardy–Weinberg equilibrium in all the groups. The rs2670660 and rs12150220 *NLRP1* SNPs as well as rs35829419 and rs10754558 *NLRP3* were not in linkage disequilibrium in all the tested groups (r^2^<0.9).

The four SNPs were not significantly different distributed in mesothelioma patients when compared to healthy controls as reported in Table 1.

**Table 1:** ***NLRP3 *and *NLRP1 *allele, genotype and haplotypes frequencies in mesothelioma patients (MM) and healthy controls (HC)**

		**MM (n=134)**	**HC (N=256)**	**P**	**OR (95% CI)**
***NLRP3 *rs35829419**	**Allele/ Genotype**				
	A	10 (0.04)	30 (0.06)		
	C	258 (0.96)	482 (0.94)	0.234	1.61 0.75-3.74
	A/A	0	0		
	C/A	10 (0.07)	30 (0.12)		
	C/C	124 (0.93)	226 (0.88)	0.221	
***NLRP3 *rs10754558**	**Allele/ Genotype**				
	G	113 (0.42)	232 (0.45)		
	C	155 (0.58)	280 (0.55)	0.405	1.14 0.83-1.55
	G/G	23 (0.17)	55 (0.21)		
	C/G	67 (0.50)	122 (0.48)		
	C/C	44 (0.33)	79 (0.31)	0.610	
**rs35829419-rs10754558**	**Haplotypes**				
	C-C	153 (0.57)	282 (0.55)	0.459	
	C-G	107 (0.40)	205 (0.40)		
	A-G	8 (0.03)	25 (0.05)		
***NLRP1 *rs2670660**	**Allele/ Genotype**				
	A	131 (0.49)	235 (0.46)		
	G	137 (0.51)	277 (0.54)	0.450	0.89 0.65-1.2
	A/A	33 (0.25)	52 (0.20)		
	A/G	66 (0.49)	131 (0.51)		
	G/G	35 (0.26)	73 (0.29)	0.604	
***NLRP1 *rs12150220**	**Allele/ Genotype**				
	A	133 (0.50)	249 (0.49)		
	T	135 (0.50)	263 (0.51)	0.821	0.95 0.71-1.31
	AA	31 (0.23)	60 (0.23)		
	AT	72 (0.54)	129 (0.50)		
	TT	31 (0.23)	67 (0.26)	0.772	
**rs2670660-rs12150220**	**Haplotypes**				
	G-T	121 (0.45)	236 (0.46)	0.588	
	A-A	118 (0.44)	205 (0.40)		
	G-A	16 (0.06)	41 (0.08)		
	A-T	13 (0.05)	30 (0.06)		

When we considered asbestos exposure, the two *NLRP3* SNPs were not associated to the development of the cancer. The NLRP1 rs2670660 G allele was more frequent in MMAE than in HCAE (0.55 versus 0.43), although not significant different after Bonferroni correction (p=0.027). The *NLRP1* rs12150220 T allele was significantly more frequent in MMAE patients (0.55) than in HCAE (0.41) (p=0.011; OR=1.79; 95%CI=1.13-2.85) (Table 2).

**Table 2:** ***NLRP3 *and *NLRP1 *allele, genotype and haplotypes frequencies in mesothelioma patients and controls exposed to asbestos (MMAE vs HCAE)**

		**MMAE (N=69)**	**HCAE (n=101)**	**p**	**OR (95% CI)**
***NLRP3 *rs35829419**	**Allele/ Genotype**				
	A	7 (0.05)	10 (0.05)		
	C	131 (0.95)	192 (0.95)	1	0.97 0.32-3.10
	A/A	0	0		
	C/A	7 (0.10)	10 (0.09)		
	C/C	62 (0.90)	91 (0.91)	1	
***NLRP3 *rs10754558**	**Allele/ Genotype**				
	G	58 (0.42)	82 (0.41)		
	C	80 (0.58)	120 (0.59)	0.823	0.94 0.59-1.50
	G/G	11 (0.16)	13 (0.13)		
	C/G	36 (0.53)	56 (0.55)		
	C/C	22 (0.31)	32 (0.32)	0.842	
**rs35829419-rs10754558**	**Haplotypes**				
	C-C	80 (0.58)	119 (0.59)	0.908	
	C-G	51 (0.37)	75 (0.37)		
	A-G	7 (0.05)	8 (0.04)		
***NLRP1 *rs2670660**	**Allele genotype**				
	A	62 (0.45)	116 (0.57)		
	G	76 (0.55)	86 (0.43)	0.027	1.65 1.04-2.62
	A/A	12 (0.18)	30 (0.30)		
	A/G	38 (0.54)	56 (0.55)		
	G/G	19 (0.28)	15 (0.15)	0.059	
***NLRP1 *rs12150220**	**Allele/ genotype**				
	A	62 (0.45)	120 (0.59)		
	**T**	**76 (0.55)**	**82 (0.41)**	**0.011**	**1.79 1.13-2.85**
	A/A	11 (0.16)	32 (0.32)		
	A/T	41 (0.59)	56 (0.55)		
	T/T	17 (0.25)	13 (0.13)	0.027	
	T/T (A/T+A/A)			0.065	2.20 0.92-5.37
	(T/T+A/T) A/A			0.030	2.43 1.08-5.84
**rs2670660-rs12150220**	**Haplotypes**				
	A-A*	**51 (0.40)**	**114 (0.56)**	0.006 *0.004	*0.52 *(0.33-0.83)
	G-T	63 (0.50)	80 (0.40)		
	G-A	6 (0.05)	6 (0.03)		
	A-T	6 (0.05)	2 (0.01)		

The *NLRP1* SNPs combined to form 4 haplotypes which showed a significantly different distribution between MMAE and HCAE (p=0.006) (Table 2). In particular the haplotype A–A SNPs was significantly less frequent in MMAE patients than in HCAE (0.40 versus 0.56; p=0.004; OR=0.52; 95% CI=0.33-0.83).

Comparing the two groups of mesothelioma patients (exposed and not exposed to asbestos), no significant difference was observed for all the 4 SNPs as reported in Table 3.

**Table 3:** ***NLRP3 *and *NLRP1 *allele, genotype and haplotypes frequencies in mesothelioma patients exposed and not exposed to asbestos (MMAE vs MMAF)**

		**MMAE (N=69)**	**MMAF (n=65)**	**p**	**OR (95% CI)**
***NLRP3 *rs35829419**	**Allele/ Genotype**				
	A	7 (0.05)	3 (0.02)		
	C	131 (0.95)	127 (0.98)	0.337	0.44 0.07-1.99
	A/A	0	0		
	C/A	7 (0.10)	3 (0.05)		
	C/C	62 (0.90)	62 (0.95)	0.327	
***NLRP3 *rs10754558**	**Allele/ Genotype**				
	G	58 (0.42)	56 (0.43)		
	C	80 (0.58)	74 (0.57)	0.902	1.04 0.62-1.74
	G/G	11 (0.16)	12 (0.19)		
	C/G	36 (0.53)	32 (0.47)		
	C/C	22 (0.31)	21 (0.34)	0.946	
**rs35829419-rs10754558**	**Haplotypes**				
	C-C	80 (0.58)	73 (0.56)	0.453	
	C-G	51 (0.37)	53 (0.41)		
	A-G	7 (0.05)	3 (0.01)		
***NLRP1 *rs2670660**	**Allele/ genotype**				
	A	62 (0.45)	70 (0.54)		
	G	76 (0.55)	60 (0.46)	0.179	1.43 0.86-2.40
	A/A	12 (0.18)	21 (0.32)		
	A/G	38 (0.54)	28 (0.44)		
	G/G	19 (0.28)	16 (0.24)	0.138	
***NLRP1 *rs12150220**	**Allele/ genotype**				
	A	62 (0.45)	71 (0.55)		
	T	76 (0.55)	59 (0.45)	0.142	1.47 0.89-2.46
	A/A	11 (0.16)	20 (0.31)		
	A/T	41 (0.59)	31 (0.48)		
	T/T	17 (0.25)	14 (0.22)	0.146	
**rs2670660-rs12150220**	**Haplotypes**				
	A.A	51 (0.40)	53 (0.41)	0.919	
	G-T	63 (0.50)	62 (0.48)		
	G-A	6 (0.05)	9 (0.07)		
	A-T	6 (0.05)	6 (0.05)		

## Discussion

Exposure to asbestos is considered the major risk factor for the onset of MM although its contribution to the pathogenesis is multifaceted and different cofactors exist [1,22]. Asbestos induces DNA alterations mostly by inducing mesothelial cells and reactive macrophages to secrete mutagenic oxygen and nitrogen species. In addition, asbestos carcinogenesis is linked to the chronic inflammatory process caused by the deposition of a sufficient number of asbestos fibres and the consequent release of pro-inflammatory molecules [23]. Genetic predisposition, radiation exposureand viral infection are co-factors that can cause MM alone or in association with asbestos and recently also with erionite, an environmental contaminant originating from the volcanic Rocks in Turkey [24].

In a previous study we have reported the presence of SV40 in 22% of MM patients from a North-Eastern Italy area massively exposed to asbestos in the past, suggesting that an association between SV40 infection and asbestos exposure, as co-morbidity factor, seem to exist [15]. Nevertheless, SV40 infection was reviled only for a small group of patients exposed to asbestos reinforcing the concept that alternative individual host factors could be involved in MM development.

In this study we considered the hypothesis of an association between known *NLRP3* single nucleotide variations and asbestos-induced mesothelioma.

NLRP3-inflammasome is known to play an important role in cancer cell transformation [25] and in the immune response against tumors [22,26] and has been described as the intracellular sensor of asbestos [19]. Thus, we hypothesized that SNPs influencing the ability to activate the inflammasome and IL-1ß secretion, could affect the response to asbestos, the entity of asbestos-induced inflammation and, finally, the predisposition to develop mesothelioma.

Data from our study did not support this hypothesis because the two SNPs analyzed in *NLRP3* gene were not found associated with mesothelioma (Table 1) or with asbestos-induced mesothelioma (Table 3). Although the two analyzed *NLRP3* SNPs were already successfully used to associate this gene to various diseases such as autoimmune (type 1 diabetes, celiac disease, SLE) [6] or infectious diseases (HIV-1) [10], it is possible that they could not be totally representative of the entire gene and deeper investigation will be needed to definitively exclude the association between *NLRP3* and asbestos-induced mesothelioma.

Nevertheless, our findings disclosed a novel hypothesis about NLR involvement in the development of mesothelioma due to asbestos. Notably, our data showed that the rs2670660 and rs12150220 polymorphisms in *NLRP1* gene were not associated to the mesothelioma. (Table 2)

This unexpected finding may suggest a predisposing effect of NLRP1 to cancer development: this could be explained considering the distribution of NLRP1 throughout the body and the unique role of NLRP1 in apoptosis. According to Kummer [14], NLRP1, and not NLRP3, is expressed in lung in both epithelial cells and alveolar macrophages where NLRP1 inflammasome possibly act as the major danger-sensing platform. The deregulation of NLRP1 inflammasome could contribute to the formation of the pro-tumor micro-environment both influencing the transformation of the lung tissue cells and/or inducing high levels of IL-1ß that contribute to growth and metastatic spread in experimental and human cancers [27-29]. The anti-apoptotic proteins Bcl-2 and Bcl-XL inhibit NLRP1 in a concentration-dependent manner [16]. It could be hypothesized that the rs2670660 G allele and rs12150220 T allele affect the level of NLRP1 expression, its function and the balance between cell surviving and apoptosis after injury influencing the mechanism of cell transformation.

Although the frequency of rs2670660 and rs12150220 polymorphism in the general population is relatively high, it does not exist any functional information about this variant. We are aware that our results are preliminary and deeper investigation will be needed to prove these pathogenic hypothesis. However we believe that this strong association between the rs12150220 polymorphism in *NLRP1* and asbestos-mesothelioma development emphasizes once more the role of inflammation and inflammasome in tumorigenesis events.

## Material and methods

### Samples

One hundred and thirty four blood samples from selected incident cases of Caucasian patients from north eastern Italy areas (Trieste, Ferrara) with malignant pleural mesothelioma were collected in the period 2009–2011 (112 males/22 females; average age 66 (SD 13) years). Of these, 69 showed an ascertained occupational asbestos exposure (MMAE) while 65 had no documented history of asbestos exposure (MMAF). In addition, blood samples were collected from 101 healthy subjects (84 males/17 females; average age 44 ± 12 yrs) from the same area with previous risk of environmental asbestos exposure (HCAE) and from 256 healthy blood donors (HC; 118 males/138 females; average age 42 ± 7) not related to the geographic area of patients’ group, were analyzed as controls.

Histological examination and classification of tumor were performed according to the World Health Organization criteria [30]. All tumor samples contained a vast majority of malignant cells and minimal stromal, inflammatory or otherwise non-malignant cells or necrotic debris. In addition, histological data on the presence of asbestos fibres (expressed as number /g of dry lung tissue) were also available (range: from 2 to 164.000 asbestos bodies).

The information about asbestos exposure, were obtained using the records of the certified Local Mesothelioma Registry, affiliated to the Italian National Mesothelioma Registry (ReNaM).

The study was approved by the Ethical Committee of the University Hospital “Ospedali Riuniti di Trieste” (Trieste, Italy).

### DNA extraction

Genomic DNA was extracted from peripheral whole blood using the EZ1 DNA purification kit (Qiagen. Milan) following manufacturer’s protocols.

### SNPs selection and genotyping

We analyzed 2 SNPs (rs35829419 and rs10754558) in *NLRP3* and 2 SNP (rs2670660 and rs12150220) in the *NLRP1* genes. SNPs genotyping was performed using commercially available TaqMan assays (Applied Biosystems. Foster City. CA). TaqMan reactions were set up based on the manufacturer’s protocol and the samples were run on the ABI7900HT Real-Time PCR platform (Applied Biosystems). Allelic discrimination was performed as suggested by the manufacturer and analyzed using the SDS software (v. 2.3) (Applied Biosystems).

### Statistical analysis

Allelic and genotypic SNP frequencies were calculated using the Genotype Transposer software [31] and then analyzed by Fisher exact test. The Haploview software [32] was used to investigate the association and linkage disequilibrium pattern and for deriving the haplotypes. The open-source R package (http://www.r-project.org) was used for Fisher exact test and odds ratio (OR) estimation. A formal Bonferroni adjustment for the number of the tests performed would require a significance threshold of p=0.013 (p_0_/N, p_0_=0.05, N=4 SNP). Dominant/recessive model has been analyzed according to Lewis [33]. Unadjusted p-values are reported in the text and tables.

## Abbreviations

MM: Malignant mesothelioma; SNP: Single nucleotide polymorphism; MMAE: Mesothelioma due to asbestos exposure; MMAF: Mesothelioma asbestos free; HC: Healthy controls; HCAE: Healthy controls exposed to asbestos; NLRP: Nod like receptor protein.

## Competing interests

The authors declare that they have no competing interests.

## Authors’ contribution

MG, SC and MC participated in conception and study design; MG, IM, RR, RB participated in collection, genetic and data analysis; MG, SC participated in statistical analysis and interpretation of the data; MG, MT and MC are involved in the preparation of the paper. All authors reviewed and approved the final paper.
